# Supplementary material for: A retrospective evaluation of access equity in virtual care during the COVID-19 pandemic: A 2-year review and comparison of visits in Ontario, Canada
Source: PLoS One. 2025 Jun 2;20(6):e0323303. doi: 10.1371/journal.pone.0323303 (PMC12129146; doi:10.1371/journal.pone.0323303)
Supplement: Attachment — Submitted filename: Response to reviewers - EVOQUE Equity - Aug 29.docx [file pone.0323303.s001.docx]

Response to reviewers

| Comment from journal | Response from authors |
| --- | --- |
| 1. It has come to our attention that the manuscript seems to be a resubmission of the manuscript PONE-D-24-28499, which was previously considered by the journal.  It is a requirement by the journal that any manuscript resubmitted after a previous round of review is accompanied by a rebuttal letter outlining the response to the original reviewers’ comments, so that the editors can establish whether the original concerns have been addressed. Can you therefore please provide us with a point-by-point response to the comments provided by the original reviewers, outlining the revisions carried out since the original submission.  We've returned your manuscript to your account. Please resolve these issues and resubmit your manuscript within 21 days. If you need more time, please email the journal office at [plosone@plos.org](mailto:plosone@plos.org). We are happy to grant extensions of up to one month past this due date. If we do not hear from you within 21 days, we will withdraw your manuscript.  Please log on to PLOS Editorial Manager at <https://www.editorialmanager.com/pone/> to access your manuscript. You will find your manuscript in the 'Submissions Sent Back to Author' link under the New Submissions menu. Be sure to remove your previous manuscript file if you are uploading a new file in response to these requests. After you've made the changes requested above, please be sure to view and approve the revised PDF after rebuilding the PDF to complete the resubmission process.  We are requesting these changes to comply with the PLOS ONE submission guidelines (<https://journals.plos.org/plosone/s/submission-guidelines>). Please note that we won't send your manuscript for review until you have resolved the above requests.  Thank you for submitting your work to PLOS ONE and supporting our mission of Open Science. | The response to the reviewers of the initial paper are found below in this response to reviewers. These were included during the last submission as well. Many thanks. |
| 1. We noticed that you did not amend the issues we have sent you. Please provide additional details regarding participant consent. In the ethics statement in the Methods and online submission information, please ensure that you have specified (1) whether consent was informed and (2) what type you obtained (for instance, written or verbal, and if verbal, how it was documented and witnessed). If your study included minors, state whether you obtained consent from parents or guardians. If the need for consent was waived by the ethics committee, please include this information.  If you are reporting a retrospective study of medical records or archived samples, please ensure that you have discussed whether all data were fully anonymized before you accessed them and/or whether the IRB or ethics committee waived the requirement for informed consent. If patients provided informed written consent to have data from their medical records used in research, please include this information  Once you have amended this/these statement(s) in the Methods section of the manuscript, please add the same text to the “Ethics Statement” field of the submission form (via “Edit Submission”).  For additional information about PLOS ONE ethical requirements for human subjects research, please refer to <http://journals.plos.org/plosone/s/submission-guidelines#loc-human-subjects-research>. | The appropriate statement was added to the “Ethics and approval” section of our manuscript. This will also be added to the ethics statement field during submission. |
| We noticed that you have not returned the Human Participants Research Checklist. Please note that the completion of this checklist is required and should it fail to be returned, your submission will be withdrawn.  Please complete all items on the Human Participants Research Checklist that are relevant for your submission.  There may be overlap between the checklist items and other queries listed below; please address any duplicated queries both in your response email and on the checklist itself. Upload the completed Human Participants Research Checklist as file type “Other” when you re-submit your manuscript. This document is for internal journal use only and will not be published if your article is accepted. The requested information will help us to assess whether your submission complies with PLOS ONE’s policies and adheres to applicable reporting standards. Note that your manuscript may be rejected if you provide incomplete or inadequate responses to the checklist questions and that changing the ‘Section/Category’ of your article does not affect this requirement.  We are requesting these changes to comply with the PLOS ONE submission guidelines (<https://journals.plos.org/plosone/s/submission-guidelines>). Please note that we won't send your manuscript for review until you have resolved the above requests. | This has been completed and will be uploaded with the manuscript. |
