# Supplementary material for: A retrospective evaluation of access equity in virtual care during the COVID-19 pandemic: A 2-year review and comparison of visits in Ontario, Canada
Source: PLoS One. 2025 Jun 2;20(6):e0323303. doi: 10.1371/journal.pone.0323303 (PMC12129146; doi:10.1371/journal.pone.0323303)
Supplement: Attachment — Submitted filename: Response to authors - Feb 2025.docx [file pone.0323303.s002.docx]

| 1. We understand that your data availability statement currently reads as follows: "The dataset from this study is held securely in coded form at ICES. While data sharing agreements prohibit ICES from making the dataset publicly available, access may be granted to those who meet pre-specified criteria for confidential access, available at [www.ices.on.ca/DAS](http://www.ices.on.ca/DAS). The full dataset creation plan and underlying analytic code are available from the authors upon request, understanding that the computer programs may rely upon coding templates or macros that are unique to ICES and are therefore either inaccessible or may require modification."  We note the following in this statement: "The full dataset creation plan and underlying analytic code are available from the authors upon request[...]"  Please be aware, PLOS’ Data Policy states that authors cannot be the sole named individuals responsible for ensuring data access (<https://journals.plos.org/plosone/s/data-availability#loc-unacceptable-data-access-restrictions>).  In light of this, please respond to the following prompts:  a.) If there are no ethical or legal restrictions, please upload the minimal anonymized data set necessary to replicate your study findings to a stable, public repository and provide us with the relevant URLs, DOIs, or accession numbers. For a list of recommended repositories, please see <https://journals.plos.org/plosone/s/recommended-repositories>. You also have the option of uploading the data as Supporting Information files, but we would recommend depositing data directly to a data repository if possible.  b.) If there are ethical or legal restrictions on sharing a de-identified data set, please explain them in detail (e.g., data contain potentially identifying or sensitive patient information, data are owned by a third-party organization, etc.) and who has imposed them (e.g., a Research Ethics Committee or Institutional Review Board, etc.). Please also provide non-author contact information for a data access committee, ethics committee, or other institutional body to which data requests may be sent. | Many thanks for this comment. This is often the case with ICES submitted papers to PLoS. We have recently submitted to PLoS Digital Health (with a different paper) and have had to justify the same elements before acceptance. (<https://shorturl.at/92nSR>)  We have amended the Data-Sharing statement to include an institutional contact for data sharing. Please see the modified manuscript for this change.  There are ethical and legal restrictions which prohibit us sharing the data with a broader audience. This secure coding is a data safety condition which is maintained between the Ministry of Health (and the data agent – ICES) as a condition of public policy research with these datasets. |
| --- | --- |

Response to reviewers
