# Supplementary material for: A retrospective evaluation of access equity in virtual care during the COVID-19 pandemic: A 2-year review and comparison of visits in Ontario, Canada
Source: PLoS One. 2025 Jun 2;20(6):e0323303. doi: 10.1371/journal.pone.0323303 (PMC12129146; doi:10.1371/journal.pone.0323303)
Supplement: Attachment — Submitted filename: Comments (2).docx [file pone.0323303.s003.docx]

All reviewer comments have been addressed appropriately. Recommend to accept for publication
